# Contingency nature of *Helicobacter bizzozeronii* oxygen-insensitive NAD(P)H-nitroreductase (HBZC1_00960) and its role in metronidazole resistance

**DOI:** 10.1186/1297-9716-44-56

**Published:** 2013-07-16

**Authors:** Pradeep Kumar Kondadi, Claudia Pacini, Joana Revez, Marja-Liisa Hänninen, Mirko Rossi

**Affiliations:** 1 Department of Food Hygiene and Environmental Health, Faculty of Veterinary Medicine, University of Helsinki, P.O. Box 66, Agnes Sjöbergin katu 2, FI-00014, Helsinki, Finland

## Abstract

Genomic analysis of a metronidazole resistant *H. bizzozeronii* strain revealed a frame length extension of the oxygen-insensitive NAD(P)H-nitroreductase HBZC1_00960 (RdxA), associated with the disruption of the C-terminal cysteine-containing conserved region (IACLXALGK). This was the result of the extension (from C_8_ to C_9_) of a simple sequence cytosine repeat (SSCR) located in the 3’ of the gene. A 3' SSCR is also present in the *rdxA* homolog of *H. heilmannii* sensu stricto, but not in *H. pylori*. We showed that in the majority of in vitro spontaneous *H. bizzozeronii* metronidazole resistant mutants, the extension of the 3′ SSCR of *rdxA* was the only mutation observed. In addition, we observed that *H. bizzozeronii* Δ*rdxA* mutant strain showed the same MIC value of metronidazole observed in the spontaneous mutants. These data indicate that loss of function mutations in *rdxA* and in particular the disruption of the conserved region IACLXALGK is associated with reduced susceptibility to metronidazole in *H. bizzozeronii*. Slipped-strand mispairing of the SSCR located in the 3′ of the *H. bizzozeronii rdxA* appears to be the main mechanism. We also observed that *H. bizzozeronii* acquires resistance to metronidazole at high mutation rate, and that serial passages in vitro without selection induced an increased level of susceptibility. In conclusion, contrary to what was previously described in *H. pylori*, the *H. bizzozeronii rdxA* appears to be a contingency gene which undergoes phase variation. The contingency nature of *rdxA* should be carefully considered when metronidazole is used in the treatment of *H. heilmannii*-associated gastritis.

## Introduction

The human-adapted pathogen *Helicobacter pylori* is one of the most common causes of bacterial infections worldwide, and it is recognized as an etiologic agent of chronic gastritis, peptic ulcers, gastric adenocarcinoma and MALT lymphoma [1]. Humans can also be sporadically infected by non-*H. pylori* gastric *Helicobacter* species, referred to as *H. heilmannii* sensu lato, that are also able to cause gastritis [2]. *H. heilmannii* s.l. comprises very fastidious zoonotic *Helicobacter* species, including *H. bizzozeronii, H. felis, H. suis* and *H. heilmannii* sensu stricto, which are all known to colonize the gastric mucosa of different animal species [2]. Although the absence of a simple laboratory test have lead to an underestimation of the infection rate, *H. heilmannii* s.l. is consider to be a rare type of zoonosis, with prevalence ranging between 0.2% and 6.5% depending of the geographic region [2-5]. Due to the rarity of these infections and the peculiar growth requirements of *H. heilmannii* s.l., which limits the isolation of pure cultures [2], very little is known about the prevalence of antibiotic resistance in these species [6-9]. Therefore, the optimal treatment regimen for these infections remains unclear, and conventional *H. pylori* eradication treatment is generally recommended [10,11]. The standard treatment for *H. pylori* appears to eradicate *H. heilmannii* s.l. infection in most patients [3,5,10-13]. However, cases of failed treatment have been reported [7,14]. The peculiar growth requirements of *H. heilmannii* s.l. have limited the application of molecular tools, affecting studies on the molecular mechanisms of antibiotic resistance in these species and hampering the adoption of new, specific strategies to treat patients after a failed treatment.

To better understand the molecular mechanisms of antibiotic resistance in *H. heilmannii* s.l., we investigated the potential reasons behind the failed treatment of a *H. bizzozeronii* infection in a 47 year-old woman suffering with chronic gastritis [7]. A few months after the diagnosis of *H. bizzozeronii*-associated gastritis, the patient was treated with a seven-day course of lansoprazole 30 mg twice daily, tetracycline 500 mg four times daily and metronidazole 400 mg three times daily. After the treatment, the patient’s symptoms became less severe and the patient started to gain weight. However, she continued to suffer from mild nausea associated with eating warm foods, and, after a few months, *H. bizzozeronii* was re-isolated from antrum samples obtained in a follow-up endoscopy [7]. The *H. bizzozeronii* obtained before the treatment was resistant to tetracycline, but a heterogeneous resistance profile for metronidazole was observed [15]. In fact, *H. bizzozeronii* CIII-1^ORG^ (an isolate obtained from the corpus of the patient‘s stomach before the treatment) showed an MIC of metronidazole equal to 32 μg/mL, but the MIC value for its derived clone CIII-1^GEN^, obtained by amplification of a single colony, was 4 μg/mL. These data indicated the simultaneous presence of metronidazole susceptible and resistant *H. bizzozeronii* variants before the treatment [15]. After treatment, the isolated *H. bizzozeronii* (antrum T_1_) was resistant to both drugs [15]. Metronidazole is considered a prodrug whose activation requires intracellular reduction by anaerobic or microaerobic microorganisms; this results in the production of bactericidal cytotoxic radicals [16,17]. In *H. pylori* the main causes of metronidazole resistance are mutations inactivating two nitroreducate genes: *rdxA* and *frxA*[16,17]. However conflicting evidence correlating the oxygen-insensitive nitroreductase RdxA and/or the NAD(P)H flavin oxidoreductase FrxA with the resistant phenotype, indicate that the molecular basis of resistance in this species remains unclear [16,17].

A comparative genomic analysis of the *H. bizzozeronii* isolates obtained before and after the treatment showed that, among the five putative nitroreductases identified in the genome of *H. bizzozeronii* CIII-1^GEN^, only the oxygen-insensitive NAD(P)H-nitroreductase HBZC1_00960, showing 47% identity with *H. pylori* RdxA HP0954, was affected [15].

This study investigates the role of HBZC1_00960 *(H. bizzozeronii* RdxA homolog) in the molecular mechanisms of metronidazole resistance in *H. bizzozeronii*.

## Materials and methods

### Bacterial strains, growth conditions, DNA manipulations and PCR

For this study, the human-derived *H. bizzozeronii* strain CIII-1^GEN^, which exhibited a metronidazole MIC of 4 μg/mL, was selected [7,15]. In addition, the canine-derived *H. bizzozeronii* CCUG 35545^T^ strain (MIC = 8 μg/mL) [18] was used for the mutation analysis. *Helicobacter* spp. strains were cultured on HP agar plates (LabM Limited, Lancashire, UK) as previously described [19]. For electroporation, *H. bizzozeronii* strains were cultivated in liquid media constituted by Brain Heart Infusion (BHI, BD, Becton, Dickinson and Co., NJ, USA) containing 10% Fetal Bovine Serum (Gibco®, Invitrogen Carlsbad, CA, USA), Skirrow selective supplement (Oxoid Ltd., Cambridge, UK) and Vitox supplement (Oxoid) (BHI-FBv) at 37°C in a jar with microaerobic atmosphere supplemented with hydrogen. The *E. coli* TOPO10 strain (Invitrogen Corporation, Carlsbad, CA, USA) was cultivated on Luria-Bertani (LB) agar or broth supplemented with 100 μg/mL ampicillin or 10 μg/mL chloramphenicol when needed. The *H. bizzozeronii* genomic DNA was prepared as previously described [20]. PCRs were performed in 25 μL reactions using Phusion® High-Fidelity DNA Polymerase (Finnzymes, Oy, Espoo, Finland) and 25 pmol of primers (Table 1).

**Table 1:** Oligonucleotides used in this study

**Oligonucleotides**	**Sequence**
HBrdxAupFW-PstI	ATTCTGCAGTGCAACACCCCAAACCCCTACACCATG
HBrdxAupRw-XbaI	CATCTAGACCACGCAGATAATCCGCATTGAGAGAACC
HBrdxAdwFW-KpnI	ATAGGTACCGGTTCTCTCAATGCGGATTATCTGCGTGG
HBrdxAdwRW-EcoRI	ATGAATTCTACTTGCTCAAAGCCACTTGATCGC
RdxAoutFw	TTCAAACGCGCCCAAGAGAGC
RdxAoutRw	GCGACCAACGCCAAGCCCAAGAC

### Antimicrobial susceptibility of *H. bizzozeronii*

The minimum inhibitory concentration (MIC) value of metronidazole (Sigma-Aldrich) was estimated using the agar dilution method. Briefly, HP agar plates supplemented with serial dilutions of the antibiotics were inoculated with 10 μL bacterial inoculums (corresponding to approximately 10^3^ cfu). The inoculum was prepared by diluting (1:10) a 0.6-0.8 OD_600_ bacterial suspension obtained from 4 days’ culture on HP agar plates. The plates were incubated at 37°C in the microaerobic incubator. The MIC values were determined by three independent assays after 4 and 6 days of incubation. *H. bizzozeronii* CIII-1^GEN^ (MIC = 4 μg/mL) was used as reference control [15]. The EUCAST 2012 clinical breakpoint of metronidazole (> 8 μg/mL) described for *H. pylori* was used to classify *H. bizzozeronii* as either resistant or susceptible [21].

### Selection of spontaneous metronidazole-resistant *H. bizzozeronii* isogenic mutants

Spontaneous metronidazole-resistant *H. bizzozeronii* mutants were selected by a single passage in media containing four times the MIC of metronidazole. Briefly, a suspension containing approximately 10^6^ cfu/mL of 3-day-grown *H. bizzozeronii* CIII-1^GEN^ was prepared in BHI-Fbv and cultured in biphasic medium (HP coupled with BHI-Fbv) for 36 h. Then, 100 μL was spread onto HP agar plates containing 16 μg/mL of metronidazole. After six to ten days of incubation, resistant colonies were picked up and transferred to a new HP plate containing 16 μg/mL of metronidazole before being frozen at −70°C in 10% glycerol for further use. From each mutant, HBZC1_00960 was amplified and sequenced. The experiment was repeated three times.

### **Construction of *H. bizzozeronii****rdxA***::*cat *(**Δ*rdxA***) isogenic mutants**

Chromosomal inactivation of the HBZC1_00960 gene (*H. bizzozeronii rdxA* homolog) was performed by allelic exchange using the chloramphenicol resistance gene (*cat*), as previously described [22]. The *cat* gene was introduced in the same direction as the target gene using *Xba*I and *Kpn*I restriction sites. The resultant plasmid, pCP5, was constructed and amplified in *E. coli* TOPO10 and used as a suicide plasmid in *H. bizzozeronii*. Mutants were obtained by electroporation as described for *H. felis*[23]. After electroporation, the bacteria were left to recover on HP agar plates for 48 h under microaerobic conditions. The mutant strains (*H. bizzozeronii rdxA*::*cat*) were selected on HP agar plates supplemented with chloramphenicol (10 mg/mL). The plates were incubated up to 10 days, and the site of recombination was verified by PCR.

### Determination of mutation rate and mutation frequency by Luria-Delbrück fluctuation analysis

The mutation rate and frequency of *H. bizzozeronii* for metronidazole were calculated using Luria-Delbrück fluctuation analysis [24]. Briefly, from a three days’ culture, a suspension of approximately 10^6^ *H. bizzozeronii* CIII-1^GEN^ per mL was prepared in BHI-Fbv broth and divided into twenty-four 0.5 mL aliquots. These aliquots were allowed to grow in biphasic medium (HP coupled with BHI-Fbv) for 36 h to obtain parallel, independent cultures. The number of resistant mutants that emerged in each culture was determined by plating an aliquot of the culture on HP agar plates containing 16 μg/mL metronidazole. The total number of cells (N_t_) was determined by plating an appropriate dilution of three cultures on non-selective medium. Colonies on both selective and non-selective plates were counted after a maximum of 10 days of incubation. The frequency of resistant mutants was expressed as the mean number of resistant cells divided by the total number of viable cells per culture. For the calculation of the mutation rate, the most likely number of mutations per culture observed (*m*_obs_) was first calculated from the distribution of numbers of resistant mutants in the independent cultures by the Ma-Sandri-Sarkar maximum-likelihood method [24] using the FALCOR web tool [25]. The effect of the sampling in the calculation of the most likely number of mutations was corrected for by applying the following equation: $m_{\mathrm{act}}=m_{\mathrm{obs}}\frac{\left(z-1\right)}{z\ln z},$ where *z* is the fraction of culture plated. Then, the mutation rate (*μ*) per cell division was calculated as: $\mu =\frac{m_{\mathrm{act}}}{N_{t}}$, where N_t_ is the total cell number per culture [24].

### Time-kill curve for metronidazole

*H. bizzozeronii* strain CIII-1^GEN^ and its derivate CIII-1^GEN^ Δ*rdxA* and CIII-1^GEN^ M11 (spontaneous metronidazole resistant mutant) were sequentially sub-cultivated. CIII-1^GEN^ was maintained on non-selective plates, CIII-1^GEN^*rdxA*::*cat* was maintained in the presence of 16 μg/mL of metronidazole, and CIII-1^GEN^ M11 was maintained in both conditions. After 10, 12 and 15 passages, approximately 10^8^ cells/mL of each *H. bizzozeronii* strain were suspended in BHI-Fbv with or without 32 μg/mL of metronidazole. After 16 h, the intracellular ATP levels were measured using BacTiter-GloTM (Promega). The experiment was performed in duplicate. The data were analyzed as percentage of relative light units (RLU) of the treated samples compared to the untreated ones. The statistical analysis was performed by applying One-way ANOVA analysis of variance followed by Tukey‘s Multiple Comparison using GraphPad Prism version 4.03 for Windows (GraphPad Software, San Diego, California, USA).

## Results

**Figure 1:**
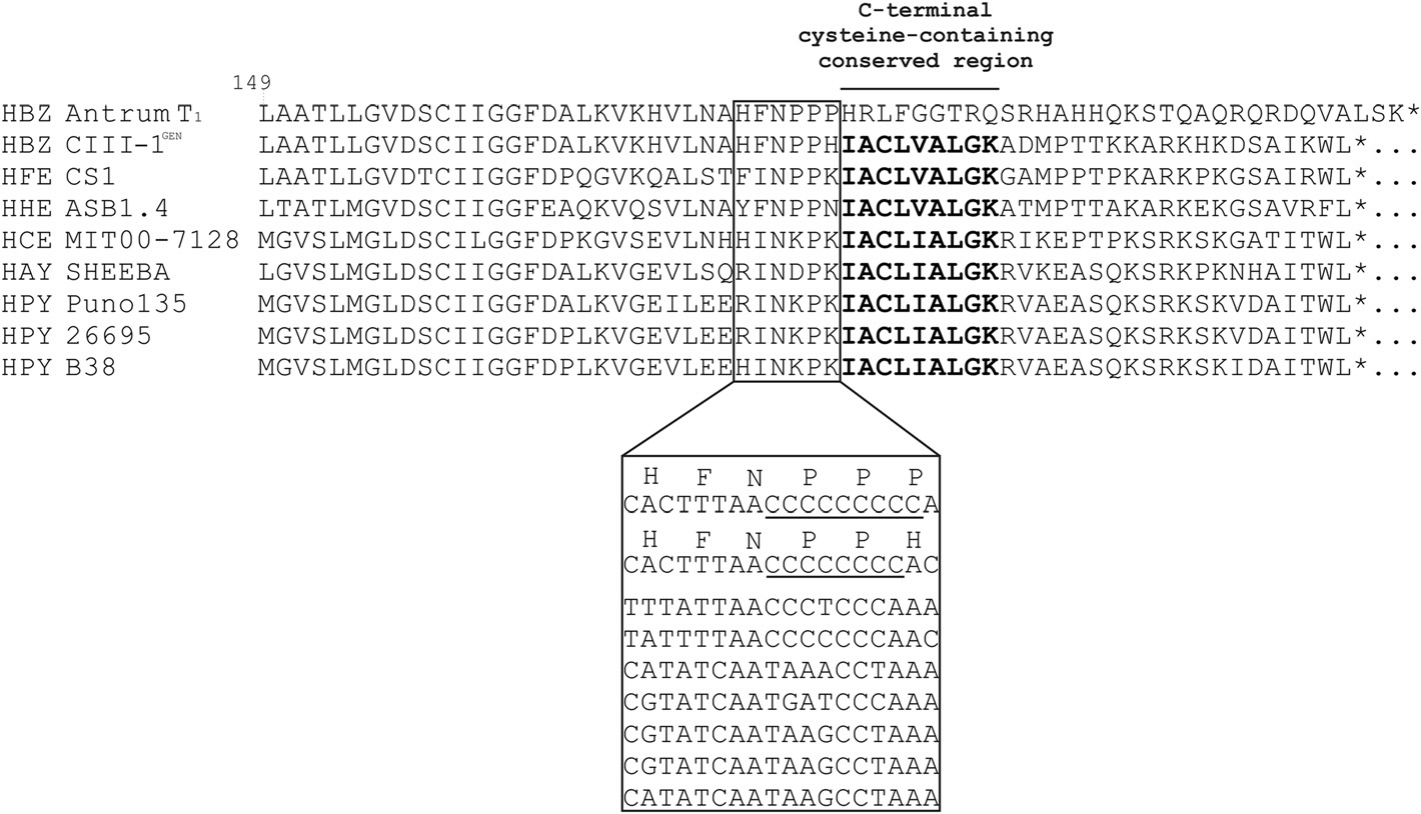
**Multialignment of the predicted amino acid sequences of RdxA C-termini from different *Helicobacter *spp.** The area where the simple sequence cysteine repeat is located in *H. bizzozeronii* is highlighted, and a multialignment of the corresponding nucleotide sequences is shown. The C-terminal cysteine-containing conserved region is marked in bold. HBZ Antrum T_1_: *H. bizzozeronii* metronidazole resistant strain (MIC 32 μg/mL); HBZ CIII-1GEN: *H. bizzozeronii* isogenic metronidazole susceptible strain (MIC 4 μg/mL); HFE CS1: *H. felis* CS1^T^ (Hfelis_12350); HHE ASB1.4: *H. heilmannii* sensu stricto ASB1.4 T (CCM10903); HCE MIT00-7128: *H. cetorum* MIT00-7128 (HCw_05595); HAY SHEEBA: *H. acinonychis* strain Scheeba (Hac_1030); HPY Puno135: *H. pylori* Puno135 (HPPN135_04725); HPY 26695: *H. pylori* 26695 (HP0954); HPY B38: *H. pylori* B38 (HELPY_0940).

A multialignment of the C-terminal part of the amino acid sequence of the RdxA homologs of *H. bizzozeronii* CIII-1^GEN^ (HBZC1_00960) and of several other *Helicobacter* species with the predicted RdxA amino acid sequence of the resistant variant *H. bizzozeronii* Antrum T_1_[15] is shown in Figure 1. As compared to the isogenic strain CIII-1^GEN^, the resistant *H. bizzozeronii* variant Antrum T_1_ showed a frame length extension leading to the disruption of the C-terminal cysteine-containing conserved region IACLXALGK (amino acids from position 182 to position 190 of HBZC1_00960) in the RdxA homolog. The frame length extension was a result of the insertion of a single cytosine in a homopolymeric run located in the C-terminal of *H. bizzozeronii rdxA* (from codon 178 to codon 180 of HBZC1_00960). A 3' simple sequence cytosine repeat (SSCR) was also present in the same position in the *rdxA* of the *H. heilmannii* s.s. type strain but not in other gastric *Helicobacter* species (Figure 1). To verify whether the instability of the 3’ SSCR of *H. bizzozeronii rdxA* was associated with metronidazole resistance, the *rdxA* genes of 11 metronidazole-resistant *H. bizzozeronii* CIII-1^GEN^ isogenic spontaneous mutants were sequenced. All of the isogenic mutants obtained in three independent experiments showed a MIC of 32 μg/mL (eight-fold more than the MIC described for the parental strain). In 10 out of 11 mutants, the extension of the SSCR (C_8_ to C_9_) in position 178–180 was the only modification observed in *rdxA*. However, the M9 mutant contained a single base deletion upstream of the SSCR, which induced the formation of a premature stop-codon in RdxA, and extension of the SSCR was not observed (Table 2).

**Table 2:** **MIC values of metronidazole after 4 days of incubation for *H. bizzozeronii *strain CIII-1**^**GEN**^**, *H. bizzozeronii *CCUG 35545**^**T**^**, and corresponding mutants**

***H. bizzozeronii *strains**	**Cytosine stretch in 3′ of *rdxA***	**Mutation**	**MIC value (μg/mL)**
CIII-1^GEN^	C_8_		4
CIII-1^GEN^ M1	C_9_		32
CIII-1^GEN^ M2	C_9_		32
CIII-1^GEN^ M3	C_9_		32
CIII-1^GEN^ M4	C_9_		32
CIII-1^GEN^ M5	C_9_		32
CIII-1^GEN^ M6	C_9_		32
CIII-1^GEN^ M7	C_9_		32
CIII-1^GEN^ M8	C_9_		32
CIII-1^GEN^ M9	C_8_	A_188_→.	32
CIII-1^GEN^ M10	C_9_		32
CIII-1^GEN^ M11	C_9_		32
CIII-1^GEN^ C1	C_8_	*rdxA::cat*	32
CIII-1^GEN^ C2	C_8_	*rdxA::cat*	32
CCUG 35545^T^	NS*		8
CCUG 35545^T^ S1	NS	*rdxA::cat*	64
CCUG 35545^T^ S2	NS	*rdxA::cat*	64

The length of the 3′ SSCR and mutations in *rdxA* are indicated for each strain.

*NS: not sequenced.

To further explore the role of *rdxA* in *H. bizzozeronii*’s resistance to metronidazole, the gene was inactivated using a chloramphenicol cassette in two different strains: CIII-1^GEN^ and CCUG 35545^T^. Due to the lack of a suitable complementation protocol for *H. bizzozeronii*, two independent mutants for each strain were selected as a control for secondary mutations. All of the mutants showed eight-fold increased MIC values for metronidazole when compared to the respective parental strain (Table 2).

The mutation rate for *H. bizzozeronii* CIII-1^GEN^ gaining metronidazole resistance was estimated by Luria-Delbrück fluctuation analysis, and the results are presented in Table 3. In the first experiment, the rate was calculated to be 4.78 × 10^-6^. However, in all of the replicates of the subsequent two experiments the number of mutant colonies was above 300, hampering the estimation of the mutation rate. Thus, in a fourth assay the fraction of plated volume (z) was decreased to 0.02, allowing the determination of a mutation rate of 1.74 × 10^-5^. The mutation frequencies were estimated to be 4.96 × 10^-5^ and 2.38 × 10^-4^ in the first and fourth experiments, respectively.

**Table 3:** **Fluctuation analysis for the calculation of the mutation frequencies and rates for *H. bizzozeronii *CIII-1**^**GEN **^**which becomes resistant to metronidazole**

**Experiment**	**No. of cells per culture (Nt)**	**Fraction of culture plated (z)**	**Resistant bacteria**			**Mutation rate (μ)**	**Mutation frequency (f)**
			**mean**	**m**_**obs**_	**m**_**act**_		
1	2.18 × 10^7^	0.08	87	22.872	104.44	4.78 × 10^-6^	4.96 × 10^-5^
2	1.07 × 10^8^	0.08	> 300	ND	ND	ND	ND
3	1.40 × 10^7^	0.08	> 300	ND	ND	ND	ND
4	1.30 × 10^7^	0.02	62	18.099	226.70	1.74 × 10^-5^	2.38 × 10^-4^

There are 21 independent cultures for each experiment.

**Figure 2:**
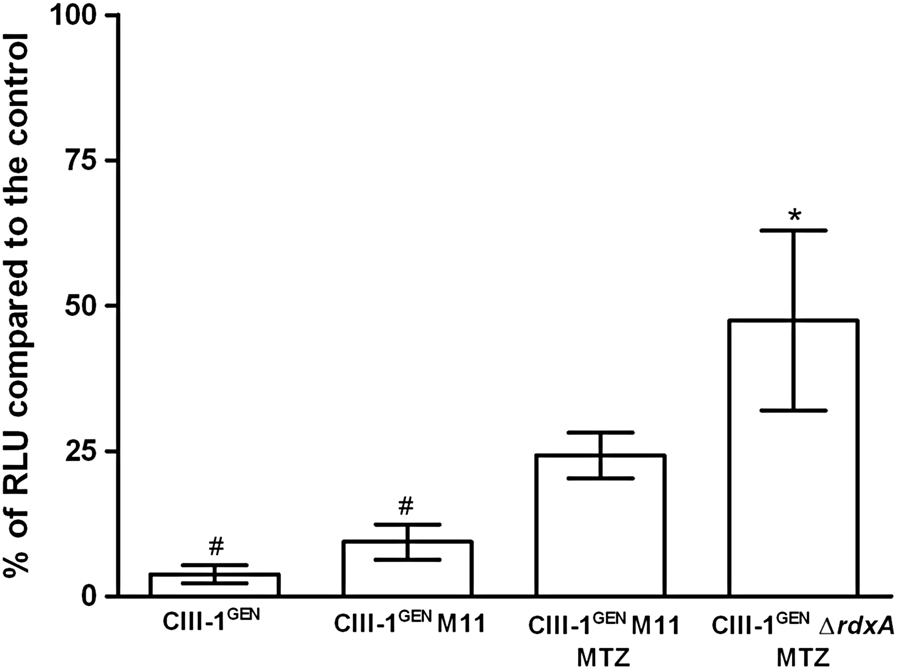
**Killing effect of metronidazole (MTZ) after 16 h of exposure against *H. bizzozeronii *strains.** The figure shows the time-kill curve for *H. bizzozeronii* strains CIII-1^GEN^, its derivate CIII-1^GEN^ Δ*rdxA* and CIII-1^GEN^ M11 maintained in the presence or absence of MTZ (marked with MTZ in the figure). Data are plotted as the percentage of intracellular ATP, shown as relative light units (RLU), of the treated samples compared to the untreated ones. Means statistically significant different from the wild type strain CIII-1^GEN^ are indicated with an asterisk, while means statistically significant different from the mutant strain CIII-1^GEN^ Δ*rdxA* are indicated with a hash tag (Turkey’s HSD, ρ < 0.05).

A luciferase-based bacterial cell viability assay was used to determine the bactericidal effect of metronidazole on *H. bizzozeronii* strain CIII-1^GEN^ and its derivate CIII-1^GEN^ Δ*rdxA* and CIII-1^GEN^ M11 after sequential subculture. Time-kill curves were generated to measure the decrease in intracellular ATP after treatment with 1× the MIC of metronidazole (calculated for the mutant *H. bizzozeronii* CIII-1^GEN^ Δ*rdxA*). The results are shown in Figure 2, where the data are plotted as a percentage of relative light units (RLU) of the treated samples compared to the untreated ones. In order to minimize the effect on the phenotype of phase variation of loci other than *rdxA*, CIII-1^GEN^ M11, obtained by pooling several colonies of spontaneous mutants from different plates, was selected. The extension of the SSCR (C_8_ to C_9_) in the position 178–180 was the only modification observed in *rdxA* of CIII-1^GEN^ M11. After 16 h of exposure, the spontaneous metronidazole-resistant isogenic mutant CIII-1^GEN^ M11 maintained on non-selective plates survived similarly to the wild type but significantly less well than CIII-1^GEN^ Δ*rdxA*. In contrast, when CIII-1^GEN^ M11 was maintained on selective plates, the percentage of survival was not significantly different to any of the other conditions tested. However, in the absence of metronidazole, CIII-1^GEN^ M11 survived less well than the same strain maintained in medium containing the antibiotic. Therefore, although no statistical significance was found between the survival capability of CIII-1^GEN^ M11 when sub-cultivated with or without metronidazole, it tends to become more susceptible in the absence of selection after a small number of in vitro passages.

## Discussion

In association with other antibiotics, metronidazole is largely used in the first-line treatment of *H. pylori* infections [26], and an increased incidence of resistant strains has been observed in the last few years [27], with current rates varying from 17% in Europe to 44% in America [28]. In contrast, almost no data are available concerning metronidazole resistance in *H. bizzozeronii* and other species of the *H. heilmannii* s.l. (such as *H. salomonis* and *H. felis*) [8]. The MICs of metronidazole for six *H. bizzozeronii* strains of animal origin have been estimated (by the agar dilution method) to range from 1 to 8 μg/mL [8], suggesting that this antibiotic could be efficiently applied to eradicate *H. bizzozeronii* infections. However, the analysis of isolates obtained from multiple biopsy samples from the same patient [7,15] revealed the simultaneous presence of metronidazole-susceptible and resistant variants [15]. These data suggest that the isogenic variation of *H. bizzozeronii* may lead to the accumulation of heteroresistant phenotypes for metronidazole, resulting in treatment failure [15].

Metronidazole resistance is a strong predictor of treatment failure for *Helicobacter* infections when the treatment contains metronidazole [26]. However, due to insufficient information about the genetic background of the resistant phenotype, non-invasive detection of metronidazole resistance is not yet feasible [16,26]. Several years of investigation have provided evidence that the main causes of metronidazole resistance in *H. pylori* are mutations that alter the correct function of the nitroreductases *rdxA* or *frxA*[16]. However, this resistance has recently been shown to involve more complex changes than the simple inactivation of *rdxA* or *frxA*, including intracellular redox potential [16] and global gene regulation [29,30]. To investigate possible changes in *H. bizzozeronii* after the acquisition of metronidazole resistance, a comparative genome analysis of susceptible and resistant isogenic strains has been performed [15]. Numerous single nucleotide polymorphisms (SNPs) and insertions or deletions (Indels) were detected in the metronidazole-resistant strain compared to the isogenic susceptible one, indicating that the modification of several genes could participate in the resistant phenotype [15]. However, among the five nitroreductases identified in the *H. bizzozeronii* CIII-1^GEN^ genome [20], only the homolog of *H. pylori rdxA* was affected in the resistant strain, and its C-terminal cysteine-containing conserved region (IACLXALGK) was disrupted [15]. This mutation resulted from the extension (from C_8_ to C_9_) of a simple sequence cytosine repeat (SSCR) located in the 3’ region of the gene. A comparative analysis showed that a similar 3' SSCR is also present in the same position in the *rdxA* homolog of *H. heilmannii* s.s. but not in *H. pylori*. In this study, we showed that the extension of the 3′ SSCR of *rdxA* was the only mutation detected in the majority of in vitro spontaneous *H. bizzozeronii* metronidazole resistant mutants. In addition, we observed that an *H. bizzozeronii* Δ*rdxA* mutant strain showed the same MIC value of metronidazole that was observed in the spontaneous mutants. These data indicate that loss of function mutations in *rdxA,* and, in particular, the disruption of the conserved region IACLXALGK, are sufficient to produce clinically significant resistance to metronidazole in *H. bizzozeronii*. These findings extend our knowledge of the types of mutations that affect the functionality of *rdxA* in *Helicobacter* spp., and they support the idea that the C-terminal cysteine-containing conserved region plays a critical role in the ability of RdxA to catalyze nitroreduction [31]. Moreover, slipped-strand mispairing of the *rdxA* 3′SSCR appears to be the most frequent mechanism leading to the inhibition of the *H. bizzozeronii* metronidazole-nitroreductase activity. Therefore, *H. bizzozeronii rdxA* represents the first example of a contingency gene being associated with metronidazole resistance.

Simple sequence repeats in *Helicobacter* and other bacterial genomes can mediate phase variation due to their high mutation rates and reversible mutations, providing a population-based mechanism for stochastic variation in expression of specific genes and rapid adaptation to environmental fluctuations [20,32]. In this study, we demonstrated that *H. bizzozeronii* acquires resistance to metronidazole at a mutation rate similar to that described for phase-variable genes in *Campylobacter jejuni* and other bacterial species [32]. In addition, we observed an increased level of susceptibility to metronidazole in a spontaneous mutant maintained in non-selective conditions after approximately 15 passages, indicating that the resistant phenotype is reversible. Based on these data, it is tempting to speculate that metronidazole resistance in *H. bizzozeronii* is a phase-variable phenotype due to the contingency nature of *rdxA*. However, several other unknown mechanisms could lead to metronidazole resistance in *H. bizzozeronii,* overcoming the stochastic variation. In addition, it is not clear how frequently the reversion of the mutation (C_9_ to C_8_) occurs. In fact, we recently detected that the larger fraction of the *H. bizzozeronii* population colonizing the stomach of a patient maintained the C_9_ allele in *rdxA* six months after the end of the therapy [15]*.* Although it is clear that disruption of the C-terminal cysteine conserved region of *rdxA* decreases the ability of *H. bizzozeronii* to catalyze the metronidazole nitroreduction, the effect of this mutation on NAD(P)H-oxidase activity is unknown. Therefore, it may be possible that the same mutation that provides resistance to metronidazole does not alter the physiological activity of *rdxA,* or paradoxically, induces an increased fitness of the bacterium. This could lead to the fixation of the mutation in the population, as observed in the patient [15].

In conclusion, *H. bizzozeronii*, and potentially other species of *H. heilmannii* s.l., easily acquired clinically significant resistance to metronidazole due to the high mutation rate of SSCR located in the 3′ region of *rdxA*. Although we observed in vitro reversibility of the phenotype, there is evidence that the mutation can be maintained in vivo even six months after the end of therapy. Therefore, the contingency nature of *rdxA* should be carefully considered when metronidazole is used in the treatment of *H. heilmannii*-associated gastritis in humans.

## Consent

Written informed consent was obtained from the patient for the publication of this report and any accompanying images.

## Abbreviations

BHI-FBv: Brain Heart Infusion containing 10% Fetal Bovine Serum, Skirrow selective supplement and Vitox supplement; LB: Luria-Bertani; MIC: Minimum inhibitory concentration; CFU: Colony forming unit; SSCR: Simple sequence cytosine repeat; RLU: Relative light unit; SNP: Single nucleotide polymorphism; Indel: Insertions or deletion.

## Competing interests

The authors declare that they have no competing interests.

## Authors’ contributions

PKK performed the experiments and helped to draft the manuscript. CP constructed the *rdxA*::cat mutants. JR participated in the design of the study and helped to draft the manuscript. MLH participated in the design of the study and helped to draft the manuscript. MR conceived and coordinated the study, designed the experiments and drafted the manuscript. All authors read and approved the final manuscript.
